# Predicting breast cancer using an expression values weighted clinical classifier

**DOI:** 10.1186/s12859-014-0411-1

**Published:** 2014-12-31

**Authors:** Minta Thomas, Kris De Brabanter, Johan AK Suykens, Bart De Moor

**Affiliations:** KU Leuven, Department of Electrical Engineering (ESAT), STADIUS Center for Dynamical Systems, Signal Processing and Data Analytics/iMinds Future Health Department, Kasteelpark Arenberg 10, Leuven, 3001 Belgium; Department of Statistics & Computer Science, Iowa State University, Ames, IA USA

## Abstract

**Background:**

Clinical data, such as patient history, laboratory analysis, ultrasound parameters-which are the basis of day-to-day clinical decision support-are often used to guide the clinical management of cancer in the presence of microarray data. Several data fusion techniques are available to integrate genomics or proteomics data, but only a few studies have created a single prediction model using both gene expression and clinical data. These studies often remain inconclusive regarding an obtained improvement in prediction performance. To improve clinical management, these data should be fully exploited. This requires efficient algorithms to integrate these data sets and design a final classifier.

LS-SVM classifiers and generalized eigenvalue/singular value decompositions are successfully used in many bioinformatics applications for prediction tasks. While bringing up the benefits of these two techniques, we propose a machine learning approach, a weighted LS-SVM classifier to integrate two data sources: microarray and clinical parameters.

**Results:**

We compared and evaluated the proposed methods on five breast cancer case studies. Compared to LS-SVM classifier on individual data sets, generalized eigenvalue decomposition (GEVD) and kernel GEVD, the proposed weighted LS-SVM classifier offers good prediction performance, in terms of test area under ROC Curve (AUC), on all breast cancer case studies.

**Conclusions:**

Thus a clinical classifier weighted with microarray data set results in significantly improved diagnosis, prognosis and prediction responses to therapy. The proposed model has been shown as a promising mathematical framework in both data fusion and non-linear classification problems.

## Background

Microarray technology, which can handle thousands of genes of several hundreds of patients at a time, makes it hard for scientists to manually extract relevant information about genes and diseases, especially cancer. Moreover this technique suffers from a low signal-to-noise ratio. Despite the rise of high-throughput technologies, clinical data such as age, gender and medical history, guide clinical management for most diseases and examinations. A recent study [1] shows the importance of the integration of microarray and clinical data has a synergetic effect on predicting breast cancer outcome. Gevaert et al. [2] have used a Bayesian framework to combine expression and clinical data. They found that decision integration, and partial integration leads to a better performance, whereas full data integration showed no improvement. These results were obtained by using a cross validation approach on the 78 samples in the van’t Veer et al. [3] data set. On the same data set, Boulesteix et al. [4] employed random forests and partial least squares approaches to combine expression and clinical data. In contrast, they reported that microarray data do not noticeably improve the prediction accuracy yielded by clinical parameters alone.

The representation of any data set with a real-valued kernel matrix, independent of the nature or complexity of data to be analyzed, makes kernel methods ideally positioned for heterogeneous data integrations [5]. Integration of data using kernel fusion is featured by several advantages. Biological data has diverse structures, for example, high dimensional expression data, the sequence data, the annotation data, the text mining data and heterogeneous nature of clinical data and so on. The main advantage is that the data heterogeneity is rescued by the use of kernel trick, where data which has diverse data structures are all transformed into kernel matrices with same size. To integrate them, one could follow the classical additive expansion strategy of machine learning to combine them linearly [6]. These nonlinear integration methods of kernels have attracted great interests in recent machine learning research.

Daemen et al. [7] proposed kernel functions for clinical parameters and pursued an integration approach based on combining kernels (kernel inner product matrices derived from the separate data types) for application in a Least Squares Support Vector Machine (LS-SVM). They explained that the newly proposed kernel functions for clinical parameter does not suffer from the ambiguity of data preprocessing by equally considering all variables. That means, a distinction is made between continuous variables, ordinal variables with an intrinsic ordering but often lacking equal distance between two consecutive categories and nominal variables without any ordering. They concluded that the clinical kernel functions represent similarities between patients more accurately than linear or polynomial kernel function for modeling clinical data. Pittman et al. [8] combined clinical and expression data for predicting breast cancer outcome by means of a tree classifier. This tree classifier was trained using meta-genes and/or clinical data as inputs. They explained that key metagenes can up to to a degree, replace traditional risk factors in terms of individual association with recurrences. But the combination of metagenes and clinical factors currently defines models most relevant in terms of statistical fit and also, more practically, in terms of cross-validation predictive accuracy. The resulting tree models provide an integrated clinico-genomic analysis that generate substantially accurate and cross-validated predictions at the individual patient level.

Singular Value Decomposition (SVD) and generalized SVD (GSVD) have been shown to have great potential within bioinformatics for extracting common information from data sets such as genomics and proteomics data [9,10]. Several studies have used LS-SVM as a prediction tool, especially in microarray analysis [11,12].

In this paper, we propose a machine learning approach for data integration: a weighted LS-SVM classifier. Initially we will explain generalized eigenvalue decomposition (GEVD) and kernel GEVD. Later we will explore the relationships of kernel GEVD with weighted LS-SVM classifier. Finally, the advantages of this new classifier will be demonstrated on five breast cancer case studies, for which expression data and an extensive collection of clinical data are publicly available.

## Data sets

Breast cancer is one of the most extensively studied cancer types for which many microarray data sets are publicly available. Among them, we selected five cases for which a sufficient number of clinical parameters were available [3,13-16]. All the data sets that we have used are available in the Integrated Tumor Transcriptome Array and Clinical data Analysis database (ITTACA). Overview of all the data sets are given in Table 1.

**Table 1:** **Summary of the 5 breast cancer data sets**

**Case study**	***#*** **Samples**		***#*** **Genes**	***#*** **Clinical variables**
	**Class 1**	**Class2**		
Case I	85	25	5000	Age, Ethnicity, ER status, PR status, Radiation treatment, Chemotherapy,
				Hormonal therapy, Nodal status, Metastasis, Tumor stage,
				Tumor size, Tumor grade.
Case II	33	96	6000	Age, Ethnicity, pretreatment tumor stage, nodal status,
				nuclear grade, ER status, PR status, HER2 status.
Case III	112	65	5000	Age, Tumor size, Nodal status, ER status, Tamoxifen treatment.
Case IV	46	51	12192	Age, Tumor size, Grade, Erp, Angioinvasion, Lymphocytic Infiltrate, PRp.
Case V	58	193	20055	Age, Tumor size, Grade, ER, Prp, Lymph node.

### Microarray data

For the first three data sets, the microarray data were obtained with the Affymetrix technology and preprocessed with MAS5.0, the GeneChip Microarray Analysis Suite 5.0 software (Affymetrix). However, as probe selection for the Affymetrix gene chips relied on earlier genome and transcriptome annotation that are significantly different from current knowledge, an updated array annotation was used for the conversion of probes to Entrez Gene IDs, lowering the number of false positives [17].

A fourth data set consists of two groups of patients [3]. The first group of patients, the training set, consists of 78 patients of which 34 patients belonged to the poor prognosis group and 44 patients belonged to the good prognosis group. The second group of patients, the test set, consists of 19 patients of which 12 patients belonged to the poor prognosis group and 7 patients belonged to the good prognosis group. The microarray data was already background corrected, normalized and log-transformed. Preprocessing step removes genes with small profile variance, less than the 10th percentile.

The last data sets consists of transcript profiles of 251 primary breast tumors were assessed by using Affymetrix U133 oligonucleotide microarrays. cDNA sequence analysis revealed that 58 of these tumors had p53 mutations resulting in protein-level changes, whereas the remaining 193 tumors were p53 wt [16].

### Clinical data

The first data of 129 patients contained information on 17 available clinical variables, 5 were excluded [13]: two redundant variables that were least informative based on univariate analysis in those variable pairs with a correlation coefficient exceeding 0.7, and three variables with too many missing values. After exclusion of patients with missing clinical information, this data set consisted of 110 patients remained in 85 of whom disease did not recur whilst in 25 patients disease recurred.

The second data in which response to treatment was studied, consisted of 12 variables for 133 patients [14]. Patient and variable exclusion as described above resulted in this data set. Of the 129 remaining patients, 33 showed complete response to treatment while 96 patients were characterized as having residual disease.

In the third data, relapse was studied in 187 patients [15]. After preprocessing, this data set retained information on 5 variables for 177 patients. In 112 patients, no relapse occurred while 65 patients were characterized as having a relapse.

The fourth data [3] consisted of predefined training and test sets same as that of corresponding microarray data. The last data set consisted of 251 patients with 6 available clinical variables [16]. After exclusion of patients with missing clinical information, this data set consisted of 237 patients of which 55 patients with p53 mutant breast tumor and the remaining patients without p53 mutant breast tumor.

## Methods

In the first section, we will discuss about GEVD and represent it in terms of ordinary EVD. Then an overview of LS-SVM formulation to kernel PCA and least squares support vector machines (LS-SVM) will be given. Next, we formulate an optimization problem for kernel GEVD in primal space and solution in dual space. Finally, by generalizing this optimization problem in terms of LS-SVM classifier, we propose a new machine learning approach for data fusion and classifications, a weighted LS-SVM classifier.

### Generalized Eigenvalue decomposition

The Generalized Singular Value Decomposition (GSVD) of *m*×*N* matrix *A* and *p*×*N* matrix *B* is [18]

(1) $$ A=U \Sigma_{A} X^{T}  $$

(2) $$ B=V \Sigma_{B} X^{T}  $$

where *U*, *V* are orthogonal matrices and columns of *X* are generalized singular vectors.

If *B*^*T*^*B* is invertible, then the GEVD of *A*^*T*^*A* and *B*^*T*^*B* can be obtained from Equations (1) and (2) as follows:

(3) $$ A^{T}A\left(X^{T}\right)^{-1}= B^{T}B\left(X^{T}\right)^{-1}\Lambda.  $$

where *Λ* is a diagonal matrix with diagonal entries $\Lambda _{\textit {ii}}=\left (\frac {\Sigma _{A_{\textit {ii}}}}{\Sigma _{B_{\textit {ii}}}}\right)^{2}$, *i*=1,…,*N*.

Equation (3) can be represented in eigenvalue decomposition (EVD) as follows:

$$ \left(B^{T}B\right)^{-1/2}A^{T}A\left(B^{T}B\right)^{-1/2}U=U \Lambda.   $$

where *U*=(*B*^*T*^*B*)^1/2^(*X*^*T*^)^−1^. The SVD of matrix *A*(*B*^*T*^*B*)^−1/2^ is given below:

(4) $$ A\left(B^{T}B\right)^{-1/2}=V \Lambda U^{T}.  $$

The matrix (*B*^*T*^*B*)^−1/2^ is defined [19] as follows: Let EVD of *B*^*T*^*B*=*TΣT*^*T*^, where columns of *T* are eigenvectors and *Σ* is a diagonal matrix. (*B*^*T*^*B*)^1/2^=*TΣ*^1/2^*T*^*T*^ and (*B*^*T*^*B*)^−1/2^=*TQT*^*T*^, where *Q* is a diagonal matrix with diagonal entries *Q*_*ii*_=(*Σ*_*ii*_)^−1/2^, *i*=1,…,*N*.

### LS-SVM formulation to Kernel PCA

An LS-SVM approach to kernel PCA was introduced in [20]. This approach showed that kernel PCA is the dual solution to a primal optimization problem formulated in a kernel induced feature space. Given training set$\{x_{i} \}_{i=1}^{N}$, $x_{i} \in \mathbb {R}^{d}$, the LS-SVM approach to kernel PCA is formulated in the primal as:

$$ \min_{w,e_{i},b} J_{p}\!\left(w,e_{i}\right)=\frac{\gamma}{2}\sum_{i=1}^{N} {e_{i}^{2}} - \frac{1}{2}w^{T}w,   $$

such that *e*_*i*_=*w*^*T*^*φ*(*x*_*i*_)+*b*, *i*=1,…,*N*, where *b* is a bias term and *φ*(.): $\mathbb {R}^{d} \rightarrow \mathbb {R}^{d_{h}}$ is the feature map which maps the *d*-dimensional input vector *x* from the input space to the *d*_*h*_-dimensional feature space.

Kernel PCA in dual space takes the form:

$$\Omega_{c}\alpha = \lambda\alpha $$

where *α* is an eigenvector, *λ* an eigenvalue and *Ω*_*c*_ denotes the centered kernel matrix with *ij*th entry: *Ω*_*c*,*i*,*j*_=$K\left (x_{i},x_{j}\right) - \frac {1}{N} \sum _{r=1}^{N} K\left (x_{i},x_{r}\right) - \frac {1}{N} \sum _{r=1}^{N} K\left (x_{j},x_{r}\right) + \frac {1}{N^{2}} \sum _{r=1}^{N} \sum _{s=1}^{N} K\left (x_{r},x_{s}\right),$ with *K*(*x*_*i*_,*x*_*j*_)=*φ*(*x*_*i*_)^*T*^*φ*(*x*_*j*_) a positive definite kernel function.

### Least squares support vector machine classifiers

A kernel algorithm for supervised classification is the SVM developed by Vapnik [21] and others. Contrary to most other classification methods and due to the way data are represented through kernels, SVMs can tackle high dimensional data (for example microarray data). Given a training set $\{x_{i},y_{i} \}_{i=1}^{N}$ with input data $x_{i} \in \mathbb {R}^{d}$ and corresponding binary class labels *y*_*i*_∈{−1,+1}, the SVM forms a linear discriminant boundary *y*(*x*)=sign[*w*^*T*^*φ*(*x*)+*b*] in the feature space with maximum distance between samples of the two considered classes, with *w* representing the weights for the data items in the feature space, *b* the bias term and *φ*(.): $\mathbb {R}^{d} \rightarrow \mathbb {R}^{n_{1}}$ is the feature map which maps the *d*-dimensional input vector *x* from the input space to the *n*_1_-dimensional feature space. This corresponds to a non-linear discriminant function in the original input space. Vapnik’s SVM classifier formulation was modified in [22]. This modified version is much faster for classification because a linear system instead of a quadratic programming problem needs to be solved.

The constrained optimization problem for least squares support vector machine (LS-SVM) [22,23] for classification are defined as follows:

$$ \min_{w,b,e} \frac{1}{2} w^{T}w+\gamma\frac{1}{2}\Sigma_{i=1}^{N} {e_{i}^{2}}   $$

subject to:

$$ y_{i}\left[w^{T}\varphi(x_{i})+b\right]=1-e_{i},\qquad i=1,\ldots,N   $$

with *e*_*i*_ the error variables, tolerating misclassifications in cases of overlapping distributions, and *γ* the regularization parameter, which allows tackling the problem of overfitting. The LS-SVM classifier formulation implicitly correspond to a regression interpretation with binary target *y*_*i*_= ±1.

In the dual space the solution is given by

$$\left[ \begin{array}{cc} 0 & y^{T} \\ y & \Omega+\frac{I}{\gamma} \end{array} \right] \left[ \begin{array}{c} b \\ \beta \end{array} \right]=\left[ \begin{array}{c} 0 \\ 1_{N} \end{array} \right] $$

with *y*=[*y*_1_,…,*y*_*N*_]^*T*^, 1_*N*_=[1,…,1]^*T*^, *e*=[*e*_1_,…,*e*_*N*_]^*T*^, *β*=[*β*_1_,…,*β*_*N*_]^*T*^, *Ω*_*i*,*j*_=*y*_*i*_*y*_*j*_*K*(*x*_*i*_,*x*_*j*_) where *K*(*x*_*i*_,*x*_*j*_) is the kernel function.

The classifier in the dual space takes the form

$$ y(x)=\text{sign} \left[\sum_{i=1}^{N} \beta_{i} y_{i} K\left(x,x_{i}\right)+b\right]   $$

where *β*_*i*_ are Lagrange multipliers.

### LS-SVM and kernel GEVD

LS-SVM formulations to different problems were discussed in [23]. This class of kernel machines emphasizes primal-dual interpretations in the context of constrained optimization problems. In this section we discuss LS-SVM formulations to kernel GEVD, which is a non-linear GEVD of *m*×*N* matrix *A*, and *p*×*N* matrix *B*, and a weighted LS-SVM classifier.

Given a training data set of *N* points $\mathcal {D} =\left \{x_{i}^{(1)},x_{i}^{(2)},y_{i}\right \}_{i=1}^{N}$ with output data $y_{i} \in \mathbb {R}$ and input data sets $x_{i}^{(1)} \in \mathbb {R}^{m}$, $x_{i}^{(2)} \in \mathbb {R}^{p}$ ($x_{i}^{(1)}$ and $x_{i}^{(2)}$ are the *i*^*th*^ sample of matrices *A* and *B* respectively).

Consider the feature maps *φ*^(1)^(.) :$ \mathbb {R}^{m}$ →$\mathbb {R}^{n_{1}}$ and *φ*^(2)^(.): $ \mathbb {R}^{p}$ →$\mathbb {R}^{n_{2}}$ to a high dimensional feature space , which is possibly infinite dimensional. The centered feature matrices $\Phi _{c}^{(1)} \in \mathbb {R}^{n_{1} \times N}$, $\Phi _{c}^{(2)} \in \mathbb {R}^{n_{2} \times N}$ become

$$ \Phi_{c}^{(1)}=\left[\varphi^{(1)}\left(x_{1}^{(1)}\right)^{T}-\hat{\mu}^{T}_{(\varphi_{1})}; \ldots; \varphi^{(1)}\left(x_{N}^{(1)}\right)^{T}-\hat{\mu}^{T}_{(\varphi_{1})}\right]^{T}   $$

$$ \Phi_{c}^{(2)}=\left[\varphi^{(2)}\left(x_{1}^{(2)}\right)^{T}-\hat{\mu}^{T}_{(\varphi_{2})}; \ldots; \varphi^{(2)}\left(x_{N}^{(2)}\right)^{T}-\hat{\mu}^{T}_{(\varphi_{2})}\right]^{T},  $$

where $\hat {\mu }_{\varphi l}= \frac {1}{N} \Sigma _{i=1}^{N} \varphi ^{(l)}\left (x_{i}^{(l)}\right)$, *l*=1,2

#### LS-SVM approach to Kernel GEVD

Kernel GEVD is a nonlinear extension of GEVD, in which data are first embedded into a high dimensional feature space introduced by kernel and then linear GEVD is applied. While considering the matrix *A*(*B*^*T*^*B*)^−1/2^ in Equation (4) and the feature maps *φ*^(1)^(.) :$ \mathbb {R}^{m}$ →$\mathbb {R}^{n_{1}}$ and *φ*^(2)^(.) :$ \mathbb {R}^{p}$ →$\mathbb {R}^{n_{2}}$ described in previous section, the covariance matrix of *A*(*B*^*T*^*B*)^−1/2^ in the feature space becomes $C \approx \Phi _{c}^{(1)} \left (\Phi _{c}^{(2)^{T}} \Phi _{c}^{(2)}\right)^{-1}\Phi _{c}^{(1)^{T}}$ with eigendecomposition *Cv*=*λv*.

While considering kernel PCA formulation based on the LS-SVM framework [24] was discussed in section ‘LS-SVM formulation to Kernel PCA’ and EVD of *Cv*=*λv* in primal space, our objective is to find the directions in which projected variables have maximal variance.

The LS-SVM approach to kernel GEVD is formulated as follows:

(5) $$ \begin{aligned} \min{_{v,e}}\; J(v,e)&= \gamma \frac{1}{2} {e}^{T} \left(\Phi_{c}^{(2)^{T}} \Phi_{c}^{(2)}\right)^{-1} e - \frac{1}{2} {v}^{T} v \\ such\; that \quad e &=\Phi_{c}^{(1)^{T}}v \;, \end{aligned}  $$

where *v* is the eigenvector in the primal space, $\gamma \in {\mathbb R}^{+}$ is a regularization constant and *e* are the projected data points to the target space.

Defining the Lagrangian

$$\mathcal{L}(v,e;\alpha)= \frac{\gamma}{2} e^{^{T}} e-\frac{1}{2} v^{^{T}} v - \alpha^{T} \left\{\left(e-\Phi_{c}^{(1)^{T}} v\right)\right\}, $$

with optimality conditions,

$$\frac{\partial \mathcal{L}}{\partial v} =0 \rightarrow v= \Phi_{c}^{(1)} \alpha $$

$$\frac{\partial \mathcal{L}}{\partial e}=0 \rightarrow \alpha=\gamma \left(\Phi_{c}^{(2)^{T}} \Phi_{c}^{(2)}\right)^{-1}e $$

$$\frac{\partial \mathcal{L}}{\partial \alpha_{i} } =0 \rightarrow {e} = \Phi_{c}^{(1)^{T}} v, $$

elimination of *v* and *e* will yield an equation in the form of GEVD

$$ \Omega_{c}^{(1)}\alpha = \lambda\Omega_{c}^{(2)} \alpha,   $$

where $\lambda =\frac {1}{\gamma }$ largest eigenvalue, $ \Omega _{c}^{(1)}$, $ \Omega _{c}^{(2)}$ are centered kernel matrices and *α* are generalized eigenvectors. The symmetric kernel matrices $\Omega _{c}^{(1)}$ and $ \Omega _{c}^{(2)}$ resolves the heterogeneities of clinical and microarray data by the use of kernel trick, where data which have diverse data structures are transformed into kernel matrices with same size.

In a special case of GEVD, if one of the data matrix is identity matrix, it will be equivalent to ordinary EVD. If $\left (\Phi _{c}^{(2)^{T}} \Phi _{c}^{(2)}\right)^{-1}=I$, then the optimization problem proposed for kernel GEVD (See Equation (5)) will be equivalent to optimization problem in [20] for the LS-SVM approach to kernel PCA.

#### Weighted LS-SVM classifier

Our objective is to represent kernel GEVD in the form of weighted LS-SVM classifier. Given the link between LS-SVM approach to kernel GEVD in Equation (5) and the weighted LS-SVM classifier (see [25] in a different type of weighting to achieve robustness), one considers the following optimization problem in primal weight space:

$$\begin{aligned} \min{_{v,e,b}}\; J(v,e)&= \gamma \frac{1}{2} e^{T} \left(\Phi_{c}^{(2)^{T}} \Phi_{c}^{(2)}\right)^{-1} e + \frac{1}{2} v^{T} v \\ such\; that \quad y&=\Phi_{c}^{(1)^{T}} v+b1_{N}+e, \end{aligned} $$

with *e*=[*e*_1_,…,*e*_*N*_]^*T*^ a vector of variables to tolerate misclassifications, weight vector *v* in primal weight space, bias term *b* and regularization parameter $\gamma \in \mathbb {R}^{+}$. Compared to the constrained optimization problem for least squares support vector machine (LS-SVM) [22,23], in this case, the error variables are weighted with a matrix $\left (\Phi _{c}^{(2)^{T}} \Phi _{c}^{(2)}\right)^{-1/2}.$

The weight vector *v* can be infinite dimensional, which makes the calculation of *v* impossible in general. One defines the Lagrangian $\mathcal {L}\left (v,e,b;\alpha \right)=\frac {1}{2} v^{T} v + \frac {\gamma }{2} e^{T} \left (\Phi _{c}^{(2)^{T}} \Phi _{c}^{(2)}\right)^{-1} e - \alpha ^{T} \left \{\left (\left (\Phi _{c}^{(1)^{T}} v\right)+b1_{N}\right)+e-y\right \},$ with Lagrange multipliers $\alpha \in \mathbb {R}^{N}.$

$$\frac{\partial \mathcal{L}}{\partial v} = 0 \rightarrow v= \Phi_{c}^{(1)} \alpha $$

$$\frac{\partial \mathcal{L}}{\partial b} = 0 \rightarrow {1_{N}^{T}} \alpha= 0 $$

$$\frac{\partial \mathcal{L}}{\partial e}=0 \rightarrow \alpha=\gamma (\Phi_{c}^{(2)^{T}} \Phi_{c}^{(2)})^{-1}e $$

$$\frac{\partial \mathcal{L}}{\partial \alpha_{i} } = 0 \rightarrow {e} + \Phi_{c}^{(1)^{T}} v+b = y $$

Elimination of *v* and *e* yields a linear system

(6) $$ \left[ \begin{array}{cc} 0 & {1_{N}^{T}} \\ 1_{N} & \Omega_{c}^{(1)} + \gamma^{-1}\Omega^{(2)}_{c} \end{array} \right] \left[ \begin{array}{c} b \\ \alpha \end{array} \right]=\left[ \begin{array}{c} 0 \\ y \end{array} \right]  $$

with *y*=[*y*_1_,…,*y*_*N*_]^*T*^, 1_*N*_=[1,…,1]^*T*^, *α*=[*α*_1_,…,*α*_*N*_]^*T*^, $\Omega ^{(1)}_{c}=\Phi _{c}^{(1)^{T}} \Phi _{c}^{(1)}$ and $\Omega ^{(2)}_{c}=\Phi _{c}^{(2)^{T}} \Phi _{c}^{(2)}.$

The resulting classifier in the dual space is given by

(7) $$ y(x)= \sum_{i=1}^{N} \alpha_{i} \left(\left[K^{(1)}(x,x_{i})+ \frac{1}{\gamma} K^{(2)}(x,x_{i})\right]+b\right)  $$

with *α*_*i*_ are the Lagrange multipliers, *γ* is a regularization parameter has chosen by user, *K*^(1)^(*x*,*z*)=*φ*^(1)^(*x*)^*T*^*φ*^(1)^(*z*), *K*^(2)^(*x*,*z*)=*φ*^(2)^(*x*)^*T*^*φ*^(2)^(*z*) and *y*(*x*) is the output corresponding to validation point *x*. The LS-SVM for nonlinear function estimation in [25] is similar to the proposed weighted LS-SVM classifier.

The symmetric, kernel matrices *K*^(1)^ and *K*^(2)^ resolve the heterogeneities of clinical and microarray data sources such that they can be merged additively as a single kernel. The optimization algorithm for the weighted LS-SVM classifier is given below:

**Algorithm: optimization algorithm for the weighted LS-SVM classifier**

1. Given a training data set of *N* points $\mathcal {D}=\left \{x_{i}^{(1)},x_{i}^{(2)},y_{i}\right \}_{i=1}^{N}$ with output data $y_{i} \in \mathbb {R}$ and input data sets $x_{i}^{(1)} \in \mathbb {R}^{m}$, $x_{i}^{(2)} \in \mathbb {R}^{p}$.
2. Calculate Leave-One-Out cross validation (LOO-CV) performances of training set with different combinations of *γ* and *σ*_1_,*σ*_2_ (bandwidths of kernel functions *K*^(1)^, *K*^(2)^) by solving linear system Equation (6) and Equation (7). In case the Leave-One-Out (LOO) approach is computationally expensive, one could replace it with a leave *p* group out strategy (*p*-fold cross-validation).
3. Obtain the optimal parameters combinations (*γ*, *σ*_1_, *σ*_2_) which have the highest LOO-CV performance.

The proposed optimization problem is similar to the the weighted LS-SVM formulation in [24] which replaced $\left (\Phi _{c}^{(2)^{T}} \Phi _{c}^{(2)}\right)^{-1}$ with a diagonal matrix to achieve sparseness and robustness.

**Figure 1:**
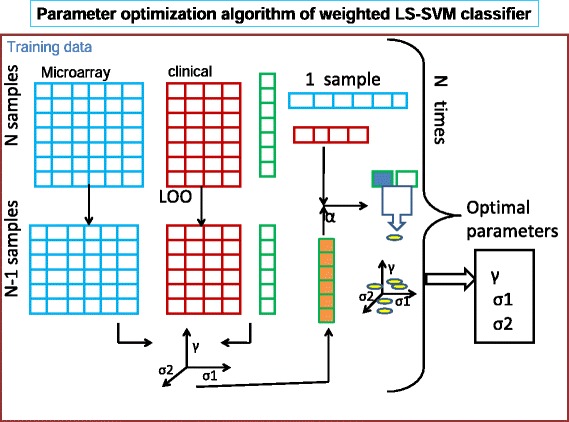
**Overview of algorithm.** The data sets represented as matrices with rows corresponding to patients and columns corresponding to genes and clinical parameters respectively for first and second data sets. LOO-CV is applied to select the optimal parameters.

The proposed method is a new machine learning approach in data fusion and subsequent classifications. In this study, the advantages of a weighted LS-SVM classifier were explored, by designing a clinical classifier. This clinical classifier combined kernels by weighting kernel inner product from one data set with that from the other data set. Here we considered microarray kernels as weighting matrix for clinical kernels. In each of these case studies, we compared the prediction performance of individual data sets with GEVD, kernel GEVD and weighted LS-SVM classifier. In kernel GEVD, *σ*_1_ and *σ*_2_ are the bandwidth of RBF-kernel function $K(x,z)=\exp \left (-\frac {||x-z||^{2}}{2 \sigma ^{2}}\right)$ of clinical and microarray data sets respectively. These parameters were chosen such that the pairs (*σ*_1_, *σ*_2_) which obtained the highest LOO-CV performance. The parameter selection (see Algorithm) for the weighted LS-SVM classifier are illustrated in Figure 1. For several possible values of the kernel parameters *σ*_1_ and *σ*_2_, the LOO cross validation performance is computed for each possible combinations of *γ*. The optimal parameters are the combinations (*σ*_1_, *σ*_2_, *γ*) with best LOO-CV performance. Remark the complexity of this optimization procedure because both the kernel parameters (*σ*_1_ and *σ*_2_) and *γ* need to be optimized in the sense of the LOO-CV performance.

## Results

In all case studies except fourth, 2/3rd of the data samples of each class are assigned randomly to the training and the rest to the test set. These randomization are the same for all numerical experiments on all data sets. This split was performed stratified to ensure that the relative proportion of outcomes sampled in both training and test set was similar to the original proportion in the full data set. In all these cases, the microarray data were standardized to zero mean and unit variance. Normalization of training sets as well as test sets are done by using the mean and standard deviation of each gene expression profile of the training sets. In the fourth data set [3], all data samples have already been assigned to a training set or test set.

Initially LS-SVM classifiers have been applied on individual data sets: clinical and microarray. Then we performed GEVD on training samples of clinical and microarray data sets and obtained generalized eigenvectors (GEV). Scores are obtained by projecting the clinical data on to the directions of GEV. LS-SVM model is trained and validated on scores corresponding to training set and test set respectively.

### Kernel GEVD

**Figure 2:**
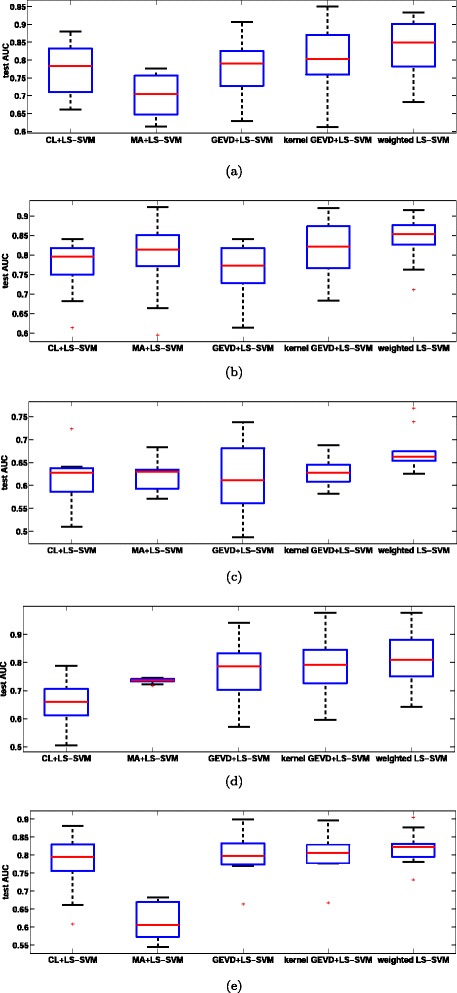
**Comparison of the prediction accuracy of the classifiers.** Boxplots of the test AUC values obtained in 100 repetitions for 5 breast cancer cases. **(a)** Case I **(b)** Case II **(c)** Case III **(d)** Case IV **(e)** Case V.

The optimal parameters of the kernel GEVD (bandwidths of clinical and microarray kernels) are selected using LOO-CV performance. We applied kernel GEVD on microarray and clinical kernels. Then we obtained the scores by projecting clinical kernels on to the direction of kernel GEV. Similar to GEVD, LS-SVM model is trained and validated on scores corresponding to training set and test set respectively. High-throughput data such as microarray have used only for the model development. The results show that considerations of two data sets in a single framework improve the prediction performance than individual data sets. In addition, kernel GEVD significantly improve the classification performance over GEVD. The results of the five case studies are shown in Table 2 and Figure 2. We represent expression and clinical data with kernel matrix, based on RBF kernel function. The RBF kernel functions makes each of the these data which has diverse structures, transformed into kernel matrices with same size.

**Table 2:** **Comparisons of different classifiers : test AUC(std) of breast cancer cases**

	**Case I**	**Case II**	**Case III**	**Case IV**	**Case V**
**Classifiers**					
CL +LS-SVM					
test AUC	0.7795(0.0687)	0.7772(0.0554)	0.6152(0.0565)	0.6622(0.0628)	0.7740(0.0833)
p-value	0.0039	1.48E-04	0.0086	5.21E-06	0.1602
MA+LS-SVM					
test AUC	0.7001(0.0559)	0.8065(0.0730)	0.6217(0.0349)	0.7357(0.0085)	0.6166(0.0508)
p-value	0.0059	0.0140	0.0254	2.41E-04	0.0020
GEVD+LS-SVM					
test AUC	0.7801(0.0717)	0.7673(0.0548)	0.6196(0.0829)	0.7730(0.1011)	0.8001(0.0648)
p-value	0.0137	3.41E-05	0.0040	0.1558	0.0840
KGEVD+LS-SVM					
test AUC	0.7982(0.0927)	0.8210(0.0670)	0.6437(0.0313)	0.7901(0.0917)	0.8031(0.0624)
p-value	0.0195	0.1144	0.0020	0.6162	0.0720
weighted LS-SVM					
test AUC	**0.8177**(0.0666)	**0.8465**(0.0480)	**0.6985**(0.0443)	**0.8119**(0.0893)	**0.8210**(0.0477)

p-value: a paired test, Wilcoxon signed rank test.

CL and MA are the clinical and microarray kernels of RBF kernel functions.

### Weighted LS-SVM classifier

We proposed a weighted LS-SVM classifier, a useful technique in data fusion as well as in supervised learning. The parameters (*γ* in Equation (6) and *σ*_1_, *σ*_2_ the bandwidths of microarray and clinical kernel functions) associated with this method are selected by Algorithm. In each LOO-CV, 1 - samples are left out and models are built for all possible combinations of parameters on the remaining *N*−1 samples. The optimization problem is not sensitive to small changes of bandwidths of microarray and clinical kernel functions. Careful tuning of *γ* allows tackling the problem of overfitting and tolerating misclassifications. Models parameters are chosen corresponding to the model with highest LOO AUC. The LOO-CV approach takes less than a minute for a single iteration of the first three case studies and 1-2 minutes for the rest of case studies. Statistical significance test are performed in order to allow a correct interpretations of the results. A non-parametric paired test, the Wilcoxon signed rank test (signrank in Matlb) [26], has been used in order to make general conclusions. A threshold of 0.05 is respected, which means that two results are significantly different if the value of the Wilcoxon signed rank test applied to both of them is lower than 0.05. On all case studies, weighted LS-SVM classifier outperformed all other discussed methods, in terms of test AUC, as shown in Table 2 and Figure 2. The weighted LS-SVM performance on second and fourth cases slightly better, but not significantly, than the kernel GEVD.

To compare LS-SVM with other classification methods, we have applied Naive Bayes classifiers individually to clinical and microarray data. In this case, the normal distribution were used to model continuous variables, while ordinal and nominal variables were modeled with a multivariate multinomial distribution. The average test AUC of this method, when applied on five case studies are shown in Table 3.

**Table 3:** **Naive Bayes classifiers performance on clinical and microarray data sets in terms of test AUC(std)**

**Data source**	**Case I**	**Case II**	**Case III**	**Case IV**	**Case V**
Clinical data	0.6235(0.0912)	0.739(0.0722)	0.5533(0.0438)	0.7156(0.0503)	0.6767(0.0513)
Microarray	0.5028(0.037)	0.6662(0.088)	0.5324(0.0616)	0.6011(0.0699)	0.5189(0.0412)

Then we compare the proposed weighted LS-SVM classifiers with other data fusion techniques which integrate microarray and clinical data sets. Daemen *et al*. [7] investigated the effect of data integration on performance with three case studies [13-15]. They reported that a better performance was obtained when considering both clinical and microarray data with the weights (*μ*) assigned to them optimized (*μ*Clinical+(1- *μ*Microarray)). In addition they concluded from their 10-fold AUC measurements that the clinical kernel variant, led to a significant increase in performance, in the kernel based integration approach of clinical and microarray. The first three case studies, we have taken from the work of Daemen *et al*. [7]. They have considered the 200 most differential genes selected from the training data with the Wilcoxon rank sum test, for the kernel matrix obtained from microarray. The fourth case study, we have taken from the paper of Gevaert *et al*. [2] in which they investigated different types of integration strategies, with Bayesian network classifier. They concluded that partial integration performs better in terms of test AUC. Our results also confirms that consideration of microarray and clinical data sets together, improves prediction performances than individual data sets.

In our analysis, microarray-based kernel matrix are calculated on large data set without preselecting genes and thus avoiding potential selection bias [27]. In addition, we compared RBF kernel with the clinical kernel function [7] on weighted LS-SVM classifier, in terms of LOO-CV performance. Results are given on Table 4. We followed the same strategy which was explained for weighted LS-SVM classifier, except the clinical kernel function have been used for the clinical parameters. On three out of five case studies, RBF kernel functions performs better than clinical kernel function.

**Table 4:** **Comparisons of RBF with clinical kernel functions in terms of LOO-CV performances**

**Kernel functions**	**Case I**	**Case II**	**Case III**	**Case IV**	**Case V**
Clinical kernel	0.8108(0.0351)	**0.8315**(0.0351)	**0.7479**(0.0111)	0.7385(0.1100)	0.7673(0.0213)
RBF	**0.8243**(0.0210)	0.8202(0.0100)	0.7143(0.0217)	**0.7846**(0.0699)	**0.7862**(0.0221)

## Discussion

Integrative analysis has been primarily used to prioritize disease genes or chromosomal regions for experimental testing, to discover disease subtypes or to predict patient survival or other clinical variables. The ultimate goal of this work is to propose a machine learning approach which is functional in both data fusion and supervised learning. We further analyzed the potential benefits of merging microarray and clinical data sets for prognostic application in breast cancer diagnosis.

We integrate microarray and clinical data into one mathematical model, for the development of highly homogeneous classifiers in clinical decision support. For this purpose, we present a kernel based integration framework in which each data set is transformed into a kernel matrix. Integration occurs on this kernel level without referring back to the data. Some studies [1,7] already reported that intermediate integration of clinical and microarray data sets improves prediction performance on breast cancer outcome. In primal space, the clinical classifier is weighted with expression values. The solution in dual space is given on Equations (6) and (7) which provides a way to integrate two kernel functions explicitly and perform further classifications.

To verify the merit of the proposed approach over the single data sources such as clinical and microarray data, the LS-SVM were built on all data sets individually for classifying cancer patients. Next, GEVD and kernel GEVD are performed. Then the projected variances in the new space (scores) have used to build the LS-SVM. Finally weighted LS-SVM approach was used for the integration of both microarray and clinical kernel functions and performed subsequent classifications. Thus weighted LS-SVM classifier proposes a new optimization framework to solve the problem of classification using features of different types such as clinical and microarray data.

We should note that the models proposed in this paper are expensive, but less than the other kernel-based data fusion techniques. Since the proposed weighted LS-SVM classifier simplified both data fusion and classification in a single framework, it does not have an additional cost for tuning parameters for kernel-based classifiers. And it is given that, the weighting matrix should be invertible in the optimization problem of kernel GEVD and the weighted LS-SVM classifier.

In life science research, there is an increasing need for heterogeneous data integration such as proteomics, genomics, mass spectral imaging and so on. Such studies are required to determine, which data sets are most significant to be considered as weighting matrix. The proposed weighted LS-SVM classifier integrates heterogeneous data sets to achieve good performing and affordable classifiers.

## Conclusion

The results suggest that the use of our integration approach on gene expression and clinical data can improve the performance of decision making in cancer. We proposed a weighted LS-SVM classifier for the integration of two data sources and further prediction task. Each data set is represented with a kernel matrix, based on the RBF kernel function. The proposed clinical classifier gives a step towards improving predictions for individual patients about prognosis, metastatic phenotype and therapy responses.

Because the parameters (bandwidth for kernel matrices and regularization term *γ* of weighted LS-SVM) had to be optimized, all possible combinations of these parameters were investigated with a LOO-CV. Since these parameters optimization strategy is time consuming, one can further investigate a parameter optimization criterion for kernel GEVD and weighted LS-SVM.

The applications of proposed method are not limited to clinical and expression data sets. Possible additional applications of weighted LS-SVM include integration of genomic information collected from different sources and biological processes. In short, the proposed machine learning approach is a promising mathematical framework in both data fusion and non-linear classification problems.
